# Greenberg Skeletal Dysplasia: first reported case in the Democratic Republic of Congo

**DOI:** 10.11604/pamj.2013.14.55.2170

**Published:** 2013-02-11

**Authors:** Toni Kasole Lubala, Nina Lubala, Arthur Ndundula Munkana, Adonis Muganza Nyenga, Augustin Mulangu Mutombo

**Affiliations:** 1 Faculté de Médecine, Université de Lubumbashi, 1825 Lubumbashi, République Démocratique du Congo; 2 Centre Interdisciplinaire de Génétique au Congo, CIGEC, Lubumbashi, République Démocratique du Congo

**Keywords:** Hydrops, skeletal dysplasia, postaxial polydactyly, Greenberg

## Abstract

We describe the first Congolese case of Greenberg Skeletal Dysplasia. Were noted at birth a congenital hydrops, a chondrodystrophy, a severe hypoplastic face as well as an ulnar (postaxial) hexadactyly on all four limbs.

## Introduction

Greenberg Skeletal Dysplasia or Hydrops-ectopic calcification-moth-eaten (HEM) is an autosomal recessive chondrodystrophy extremely rare characterized by fetal hydrops, short limbs and abnormal chondro-osseous calcification. Since Greenberg described the first case in 1988 [1], only about ten other cases had been reported in the literature [2]. In this article, we describe the first Congolese case of Greenberg skeletal dysplasia.

## Patient and observation

NN, a female new-born of caesarean section delivery at 36 weeks gestation weighted at birth 2700g, her height was 44cm and her head circumference 35 cm. She was born in Lubumbashi in the South of the Democratic Republic of the Congo (D.R.C). Her mother is a 41 years old multiparous, grava 12 para 11. Both her parents are Congolese but non consanguineous and there was no relevant family history.

**Figure 1:**
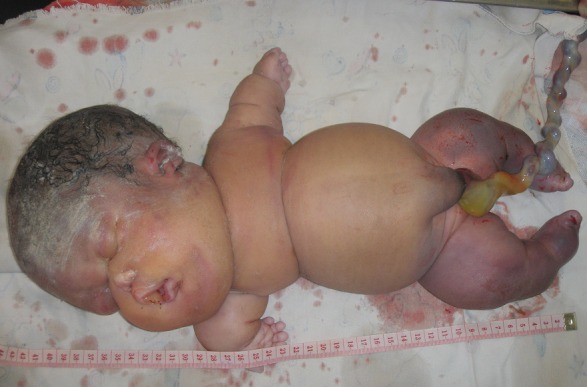
The new-born presented an hydrops fetalis, a narrow thorax and a protuberant abdomen

**Figure 2:**
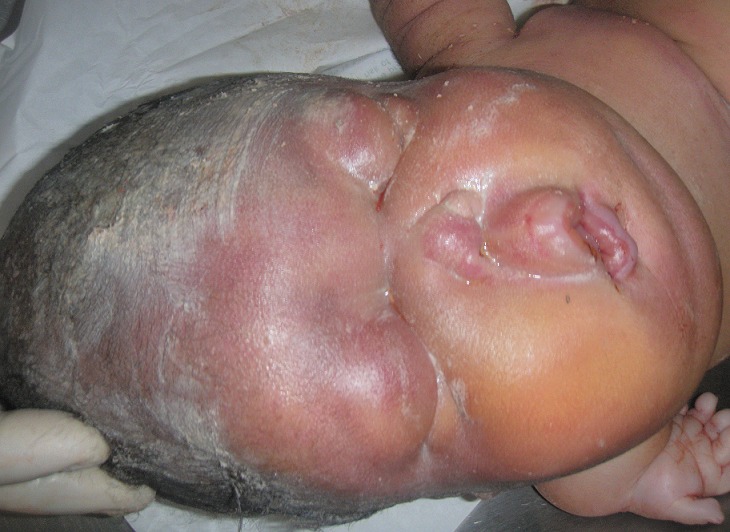
Note the important hypoplastic face

**Figure 3:**
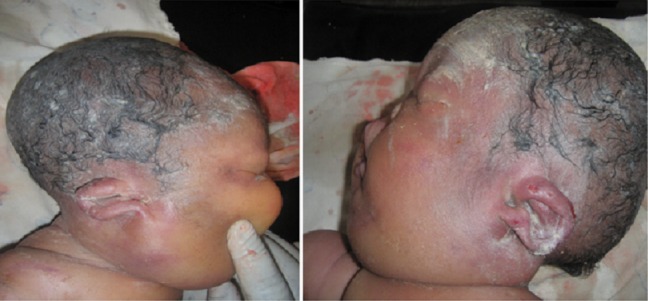
A bilateral microtia with ears set low on the head

**Figure 4:**
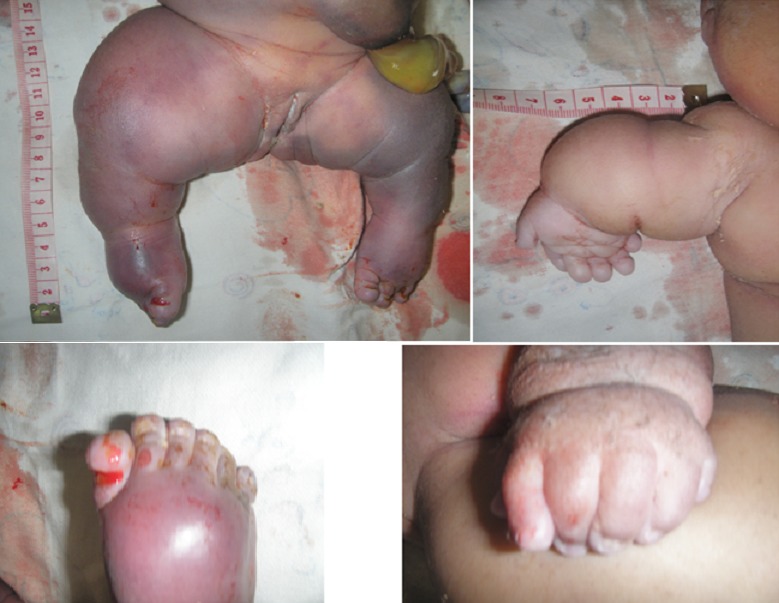
Short-limbed dwarfism was observed: short arms and forearms as well as short thighs and legs

The clinical examination revealed that NN was born alive and presented a severe hydropsfetalis (Figure 1). Were also noted an important hypoplastic face (Figure 2), a bilateral microtia with ears set low on the head (Figure 3). The new-born presented also a narrow thorax and a protuberant abdomen (Figure 1). Short-limbed dwarfism was observed (Figure 4): short arms and forarms (upper limbs: 11cm) as well as short thighs and legs (lower limbs 14 cm). The hand examination revealed a brachydactyly and a postaxial (ulnar) hexadactyly on all four limbs (Figure 4). The patient died at day 2 before a radiographic examination of the entire skeleton and an abdominal ultra-sound had been performed.

## Discussion

Greenberg skeletal dysplasia is an autosomal recessive syndrome extremely rare [3]. This syndrome was described within different ethnic groups and particularly in new-borns with consanguineous parents. Until now, no other case has been reported in the D.R.C, a big country with more than 400 ethnic groups where endogamic marriages are observed only in few of these tribes, the Lunda tribe for example, being one of them. In the case we describe, the female new-born had nonconsanguineous Congolese parents and this is similar to the cases described by Horn et al in 2003 [3] in Germany and by Trajkovski et al [4] in Macedonia. Waterham et al. works, published in 2003 have proved that genetically, Greenberg dysplasia is associated with an inherited disorder of cholesterol biosynthesis caused by LBR gene mutations that lead to the loss of the sterol reductase function of the lamin B receptor [5]. Our patient&apos;s clinical examination revealed a severe hydropsfetalis, a short-limbed dwarfism (upper limbs: 11 cm and lower limbs: 14 cm) and a brachydactyly. Was also observed a postaxial hexadactyly on all four limbs like in the case described by Chitaya et al [6]. A narrow thorax and a protuberant abdomen were observed (Figure 3) as well as a microtia with the ears set low on the head. We were not able to perform different radiographs of the skeleton which might have helped us identify ectopic ossification and/or dysplaseal dysplasia as reported in the other cases of Greenberg skeletal dysplasia [1]. An abdominal ultrasound that was not performed either, may have been done to look for an ascites or an intestinal malrotation [3]. This syndrome has a lethal course in most of the reported cases as well as in the first case reported by Greenberg et al. who died in-utero. In our case, the patient was born alive and died two days later.

## Conclusion

We have described the first Congolese case of Greenberg skeletal dysplasia, an extremely rare genetic syndrome with only about ten cases to be reported in medical literature up until 2009.
